# Supplementary material for: Correlation of C-arm CT acquired parenchymal blood volume (PBV) with 99mTc-macroaggregated albumin (MAA) SPECT/CT for radioembolization work-up
Source: PLoS One. 2020 Dec 30;15(12):e0244235. doi: 10.1371/journal.pone.0244235 (PMC7773241; doi:10.1371/journal.pone.0244235)

***S1 Data***

**S1 Table**. Size, PBV, TBR and MAA-uptake of all measured tumor lesions.

| **S2 Table: Log-transformed PBV and ^99m^Tc-MAA tumor-to-background ratio as well as ^99m^Tc-MAA-uptake of tumor lesions (U_lesion_) and liver background (U_background_).** Mean ± standard deviation is given with 95%CI in brackets. | | | | | | | |
| --- | --- | --- | --- | --- | --- | --- | --- |
|  | | |  | | | |  |
|  |  | **C-arm CT** | **^99m^Tc**-**MAA-SPECT/CT** | | | | **n** |
|  |  |  |  |  |  | |  |
|  |  | **ln(PBV_lesion_)** [ml/100 ml] | **ln(MAA-TBR)** | **ln(U_lesion_)** [kBq/cm^3^] | **ln(U_background_)** [kBq/cm^3^] | |  |
| **Average** |  | 2.2 ± 0.8  (2.0-2.3) | 1.5 ± 0.7  (1.3-1.6) | 3.9 ± 0.9  (3.7-4.0) | 2.4 ± 0.8  (2.3-2.6) | | 102 |
| **Tumor entity** | |  |  |  |  | |  |
| HCC | | 2.4 ± 0.7  (2.2-2.6) | **1.7 ± 0.8^+^**  **(1.4-1.9)** | 4.0 ± 1.1  (3.6-4.3) | 2.3 ± 1.0  (2.0-2.6) | | 48 |
| ▪ capsular | | 2.1 ± 0.7  (1.9-2.2) | 1.4 ± 0.7  (1.3-1.6) | 3.8 ± 0.8  (3.6-4.0) | 2.4 ± 0.8  (2.2-2.6) | | 31 |
| ▪ diffuse | | 2.5 ± 0.8  (2.1-2.8) | 1.7 ± 0.8  (1.3-2.0) | 4.3 ± 0.8  (4.0-4.7) | 2.7 ± 0.8  (2.3-3.1) | | 17 |
| CRC | | 2.0 ± 0.7  (1.8-2.2) | **1.2 ± 0.5^+^**  **(1.1-1.4)** | 3.8 ± 0.6  (3.6-4.0) | 2.6 ± 0.6  (2.4-2.7) | | 54 |
| ▪ capsular | | 1.9 ± 0.7  (1.6-2.2) | 1.4 ± 0.4  (1.2-1.5) | 3.6 ± 0.6  (3.4-3.9) | 2.3 ± 0.6  (2.1-2.5) | | 23 |
| ▪ diffuse | | 2.0 ± 0.7  (1.8-2.3) | 1.2 ± 0.5  (1.0-1.4) | 3.9 ± 0.7  (3.7-4.2) | 2.8 ± 0.5  (2.6-2.9) | | 31 |
| **Growth pattern** | |  |  |  |  | |  |
| ▪ capsular | | 2.2 ± 0.7  (2.0-2.4) | 1.6 ± 0.7  (1.4-1.7) | 3.7 ± 1.0  (3.5-4.0) | **2.2 ± 0.9°**  **(1.9-2.4)** | | 54 |
| ▪ diffuse | | 2.2 ± 0.8  (2.0-2.4) | 1.3 ± 0.7  (1.2-1.5) | 4.1 ± 0.7  (3.9-4.3) | **2.7 ± 0.6°**  **(2.5-2.9)** | | 48 |
| **Tumor size** | |  |  |  |  | |  |
| ≤ 25 mm | | 2.3 ± 0.8  (2.1-2.5) | 1.5 ± 0.7  (1.3-1.7) | 3.9 ± 0.9  (3.7-4.2) | 2.5 ± 0.9  (2.2-2.8) | | 46 |
| > 25 mm | | 2.1 ± 0.7  (1.9-2.2) | 1.4 ± 0.7  (1.3-1.6) | 3.8 ± 0.8  (3.6.3-4.0) | 2.4 ± 0.8  (2.2-2.6) | | 56 |
| Significant differences of log-transformed data are marked bold. Significant effects were found for ln(MAA-TBR)^(+)^ between HCC and CRC (p^(+)^=0.03) and for ln(U_background_) in the liver in case of capsular or diffuse tumor growth (p^(^°^)^= 0.022) | | | | | | | |
|  | | | | | |  |  |

**S1a Fig:** Distribution of log normal transformed TBR


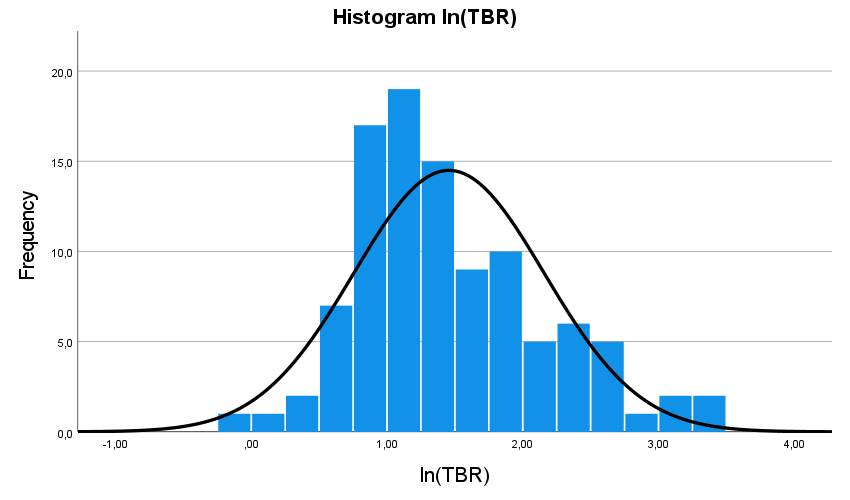


**S1b Fig:** Distribution of log normal transformed PBV


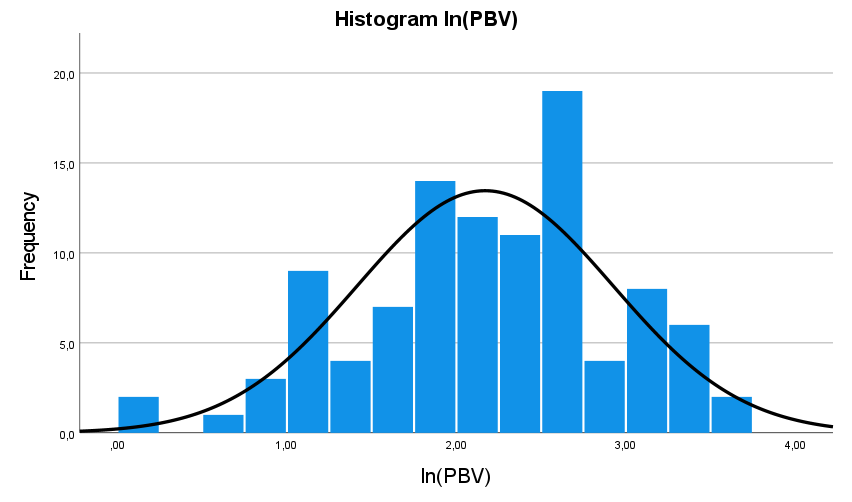


**S1c Fig:** Distribution of log normal transformed tumor size measured in PBV maps.


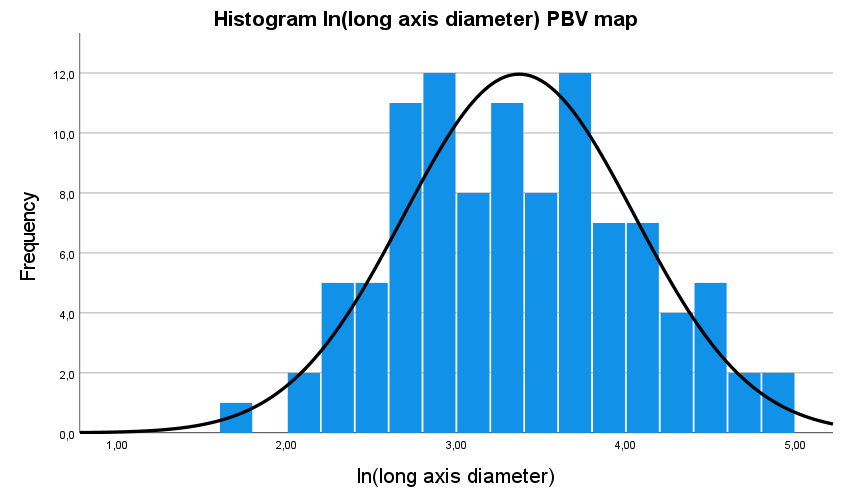


**S1d Fig:** Distribution of log normal transformed tumor size measured in MAA-SPECT/CT.

**
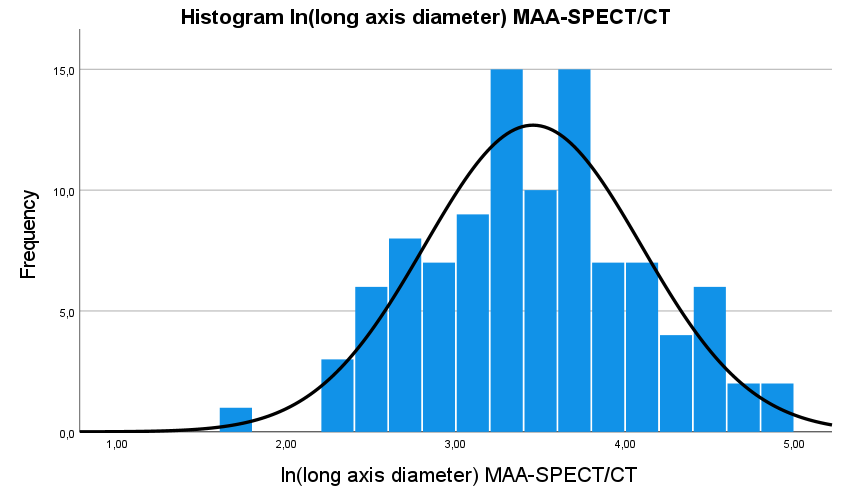
**

**S2 Fig.** Scatterplott of PBV values in dependency of semi-quantitative measured MAA-uptake.


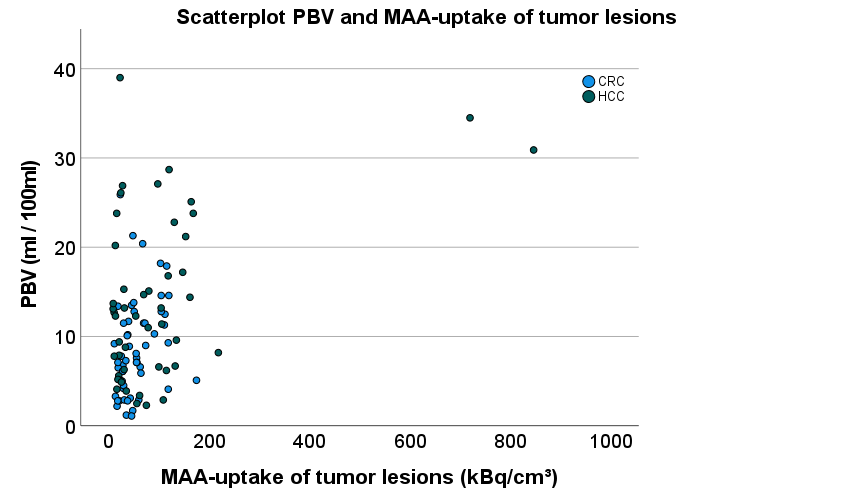

Supplement: S1 Data — (DOCX) [file pone.0244235.s001.docx]
